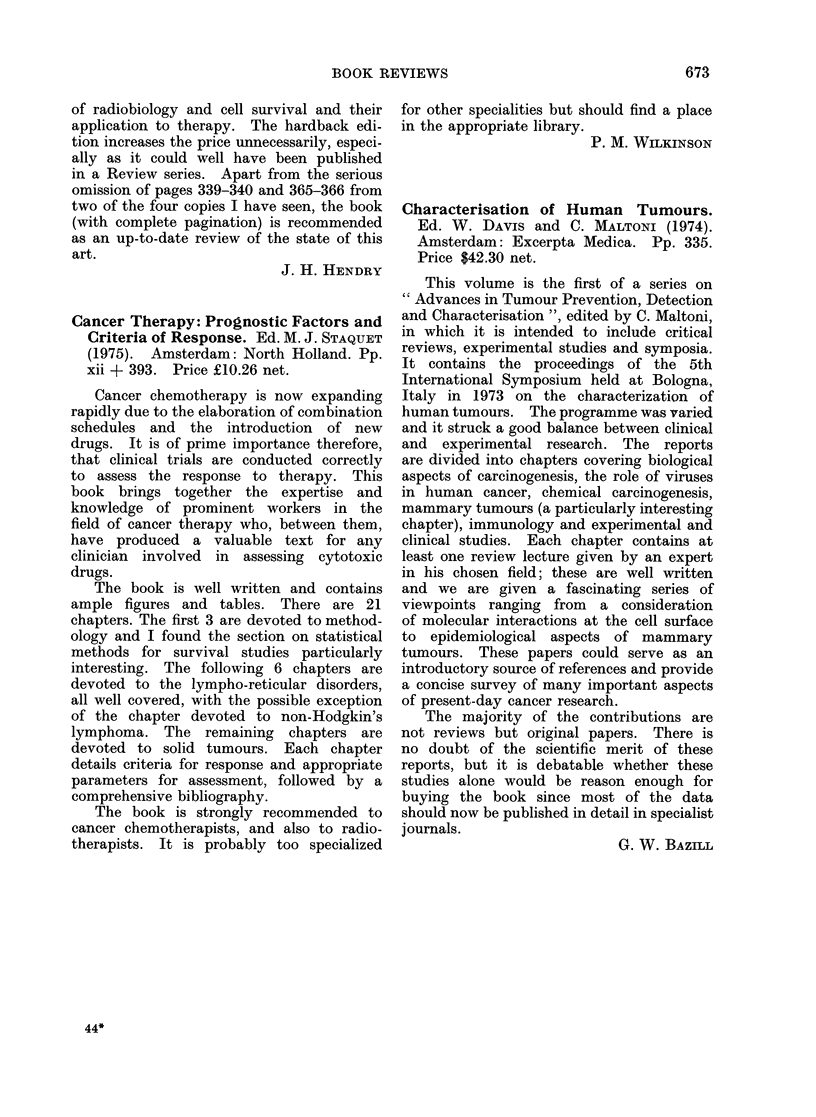# Characterisation of Human Tumours

**Published:** 1976-06

**Authors:** G. W. Bazill


**Characterisation of Human Tumours.** Ed. W. DAVIS and C. MALTONI (1974). Amsterdam: Excerpta Medica. Pp. 335. Price $42.30 net.

This volume is the first of a series on " Advances in Tumour Prevention, Detection and Characterisation ", edited by C. Maltoni, in which it is intended to include critical reviews, experimental studies and symposia. It contains the proceedings of the 5th International Symposium held at Bologna, Italy in 1973 on the characterization of human tumours. The programme was varied and it struck a good balance between clinical and experimental research. The reports are divided into chapters covering biological aspects of carcinogenesis, the role of viruses in human cancer, chemical carcinogenesis, mammary tumours (a particularly interesting chapter), immunology and experimental and clinical studies. Each chapter contains at least one review lecture given by an expert in his chosen field; these are well written and we are given a fascinating series of viewpoints ranging from a consideration of molecular interactions at the cell surface to epidemiological aspects of mammary tumours. These papers could serve as an introductory source of references and provide a concise survey of many important aspects of present-day cancer research.

The majority of the contributions are not reviews but original papers. There is no doubt of the scientific merit of these reports, but it is debatable whether these studies alone would be reason enough for buying the book since most of the data should now be published in detail in specialist journals.

G. W. BAZILL